# Knockdown of *Thitarodes* host genes influences dimorphic transition of *Ophiocordyceps sinensis* in the host hemolymph

**DOI:** 10.3389/fcimb.2024.1451628

**Published:** 2024-09-27

**Authors:** Tanqi Sun, Yongling Jin, Zhongchen Rao, Wang Liyan, Rui Tang, Khalid Muhammad Zaryab, Mingyan Li, Zhenhao Li, Ying Wang, Jing Xu, Richou Han, Li Cao

**Affiliations:** ^1^ College of Agriculture, Heilongjiang Bayi Agricultural University, Daqing, China; ^2^ Guangdong Key Laboratory of Animal Conservation and Resource Utilization, Guangdong Public Laboratory of Wild Animal Conservation and Utilization, Institute of Zoology, Guangdong Academy of Sciences, Guangzhou, China; ^3^ Research Centre, Zhejiang Shouxiangu Pharmaceutical Co. Ltd, Zhejiang, Jinhua, China; ^4^ Research Centre, Zhejiang Yuewangshengcao Biotechnological Company Limited, Zhejiang, Jinhua, China

**Keywords:** Chinese cordyceps, *Ophiocordyceps sinensis fungus*, *Thitarodes/Hepialus* ghost moth, blastospore-mycelium transition, RNAi

## Abstract

The Chinese cordyceps, a unique parasitic complex of *Thitarodes*/*Hepialus* ghost moths and *Ophiocordyceps sinensis* fungus in the Tibetan Plateau, is a highly valuable biological resource for medicine and health foods in Asian countries. Efficient system for artificial cultivation of Chinese cordyceps relies on understanding the gene functions involved in the induction of growing blastospores into hyphae in the larval hemolymph of insect host, during *O*. *sinensis* infection. Transcriptome analysis and ribonucleic acid interference (RNA interference) method were employed to identify the key differentially expressed genes and to demonstrate their functions in *Thitarodes xiaojinensis*. Key larval genes critical for *O*. *sinensis* blastospore development or filamentation were identified. Nine of the 20 top upregulated genes encoded cuticles proteins, indicating that these proteins highly activated when the larval hemolymph was full of blastospores. Small interfering RNA (siRNA) knockdown of five larval genes such as *Flightin*, *larval cuticle protein LCP-30*, *26-hydroxylase* (*CYP18A1*), *cuticle protein 18*.*6*, *isoform B*, and *probable chitinase 3* significantly stimulated the dimorphic transition from blastospores to prehyphae in *O*. *sinensis* in the larval hemolymph after 120 h after injection. The expressions of these genes determined by quantitative real-time PCR were suppressed in various levels from 38.64% to 91.54%, compared to the controls. These results demonstrated that injection of the siRNAs of key upregulated genes into the larval hemolymph containing high load of blastospores caused the gene silence in *T*. *xiaojinensis* larvae and induced the fungal transition from blastospores to prehyphae, providing novel knowledge on the regulation of *O*. *sinensis* fungal dimorphism by *Thitarodes* host and cues for further study of *Thitarodes* biology and commercial cultivation of Chinese cordyceps.

## Introduction

The Chinese cordyceps is a unique parasitic complex formed by the parasitisation of *Thitarodes Hepialus* spp. (Hepialidae, Lepidoptera) by *Ophiocordyceps sinensis* fungus in the Qinghai-Tibet Plateau, usually between 3,000 and 5,000 m (Li et al., 2011; Xia et al., 2015), and regarded as a highly valuable biological resource for medicines and health foods in the Eastern Asian countries (Yue et al., 2013a; Li et al., 2016; Baral, 2017; Pouliot et al., 2018; Qin et al., 2018). Global climate warming, overexploitation, and limited distribution greatly reduce the annual yield of Chinese cordyceps in nature (Liang et al., 2008), resulting in the list of an endangered species [The Convention on International Trade in Endangered Species Wild Fauna and Flora (CITES-II)] for protection (by CITES Management Authority of China and China Customers, 2000). However, the market demand for Chinese cordyceps is still increasing sharply in many countries (Paterson, 2008; Shrestha and Bawa, 2014), due to the increasing discovery of its medicinal functions. The wild fungus–insect complex costs $60,000–$75,000 per kilogram and is used to treat a variety of ailments, including impotence, fatigue, and cancer (Holliday and Cleaver, 2008; Yue et al., 2013b; Han et al., 2019).

Artificial cultivation of Chinese cordyceps provides a great potential to ensure its protection as a bio-resource and for commercial supply. During the artificial cultivation process, the formation of Chinese cordyceps mainly contains five critical stages: infection of *Thitarodes*/*Hepialus* larvae by ascospores, blastospores (Liu et al., 2019, 2020), or conidia of *O*. *sinensis* fungus (Wu et al., 2022), growth of blastospores in the hemolymph of the infected living larvae; blastospore transition to hyphae under unknown specific conditions in the hemolymph; mycelial mumification of the larval body; and production of stromata and fruiting body from the larval cadaver (Meng et al., 2015; Rao et al., 2019a; Wu et al., 2020). The dimorphic transition from blastospores to hyphae is considered to be the most critical step during Chinese cordyceps production.

Dimorphism is a common phenomenon in fungi, especially in pathogenic fungi, which enables fungi to adapt and colonize new environmental niches (Nemecek et al., 2008; Gauthier, 2015; Liu et al., 2019; Ruiz-Herrera et al., 2020). Unicellular yeast-like form and multicellular hyphae exhibit in these dimorphic fungi. The yeast-like to hypha transition can be triggered by many nutritional and environmental factors, such as nutrient starvation (Naseem et al., 2011), neutral pH (Jürgensen et al., 2001), temperature (Bastidas and Heitman, 2009; Gao et al., 2022), serum, and molecules that contribute to quorum sensing (Albuquerque and Casadevall, 2012; Berrocal et al., 2012), in other fungal systems. The mitogen-activated protein kinase cascade, protein kinase A (PKA), Snf1, and target of rapamycin pathways (Chaves et al., 2016; Su et al., 2018; Gomez-Gil et al., 2019) have been associated with this morphotype transition. *O*. *sinensis* fungus also exhibits blastospore-hypha dimorphism (Liu et al., 2019, 2020). The larvae infected by *O*. *sinensis* can survive for several months before mummification (Meng et al., 2015; Rao et al., 2019b), indicating that *Thitarodes* insect hosts should be involved in blastospore-hypha transition *in vivo*.

In fact, the functional genes or pathways are reported from ghost moth *Thitarodes* insects, on the basis of the transcriptome analysis. Immunity-related transcripts in *T*. *xiaojinensis* are identified in a rapid response to *O*. *sinensis* challenge, but this insect species develops tolerance to the fungus after prolonged infection by immune suppression (Meng et al., 2015). Differentially expressed genes (DEGs) are detected in *T*. *pui* larvae in low or high altitude (Wu et al., 2015). Genes encoding physical barriers such as cuticle proteins and peritrophic matrix proteins, immune response such as antimicrobial peptides (AMPs) and pattern recognition receptors, and enzymes in the proteolytic cascade are predicted to be involved in the response of *T*. *jiachaensis* to *O*. *sinensis* infection after 15 days (Li et al., 2016) and AMP repertoire (such as cecropin, defensin, attacin, and gloverin) of *T*. *armoricanus* responding to *Escherichia coli* challenge after 72 h (Wang and Hu, 2017). *T*. *armoricanus* adopts three common strategies for adaptation to hypoxia or anoxia: upregulated signal transduction pathways essential for cellular survival, strengthened cell and organelle structure and activity, and activated immune system (Rao et al., 2019b). After 3 and 15 days, the genes in osmotic imbalance, immunocompromise (such as *DEFs* and *GLVs*), and nervous system dysfunction (glutamatergic synapse) may be involved in the rapid death of *T*. *armoricanus* after *Paecilomyces hepiali* infection, and upregulation of the genes related to cuticle structure, nervous system (such as neurotrophin signal pathway and dopaminergic synapse), and immune effectors (such as *attacin* and *proline-rich antimicrobial peptide 1*) may contribute to the co-existence of *T*. *armoricanus* and *O*. *sinensis* (Rao et al., 2019b). DEGs for the aggressive behavior of *T*. *xiaojinensis* toward conspecifics and heterospecifics are also analyzed to provide a theoretical basis for the further analysis of the genetic mechanism of the insect’s aggression (Rao et al., 2021). However, a global comparison of the genes of ghost moth larvae responsible for hyphal conversion from blastospores in *O*. *sinensis* and for the mummification of the infected larvae is still lacking.

To explore the possible involvement of the genes and pathways from the larvae of *T*. *xiaojinensis* in the regulation of mycelial transition from blastospores, in this study, high-throughput RNA sequencing (RNA-seq) was utilized to analyze time-course transcriptome profiles of *T*. *xiaojinensis* larvae infected with *O*. *sinensis*, in three stages, including living larvae with low blastospore load (less than 20 blastospores per 10 μL of hemolymph) (BL) and with high blastospore load (more than 200 blastospores per 10 μL of hemolymph) (BH). Several important pathways and key genes from *Thitarodes* larvae critical for *O*. *sinensis* blastospore development or filamentation were identified. siRNA knockdown of key larval genes in the hemolymph significantly stimulated the dimorphic transition from blastospores in stationary stage to prehyphae, pod-like multinuclear unbranched segmented filaments (Li et al., 2020), of *O*. *sinensis* fungus. The results provide novel knowledge on the regulation of *O*. *sinensis* fungal dimorphism by *Thitarodes* host and cues for further study of *Thitarodes* biology and commercial utilization of Chinese cordyceps.

## Materials and methods

### Insects

*T*. *xiaojinensis* was reared in the low-altitude laboratory according to the described methods (Tao et al., 2015). Briefly, the ghost moth pupae were kept at 9°C–17°C and 50%–80% relative humidity in plastic containers (L = 50 cm, W = 40 cm, and H = 30 cm) with moist moss. The emerged adults were housed in equal proportions of males and females in small mosquito nets (L = 104 cm, W = 50 cm, and H = 50 cm) for mating and oviposition. The hatched larvae were transferred to a culture room at 9°C–13°C and offered the roots of *Potentilla anserina* as food to obtain fifth instar larvae (average fresh weight = 0.54 ± 0.04 g) for fungal infection. *T*. *xiaojinensis* was identified by using the amplified Cytochrome b sequence with the primers CB1 (TATGTACTACCATGAGGACAAATATC) and CB2 (ATTACACCTCCTAATTTATTAGGAAT (Tao et al., 2015; Liu et al., 2016).

### Fungal isolate

The KD1223 isolate of *O*. *sinensis* preserved at −80°C was cultured on Peptone Potato Dextrose Agar (PPDA) medium (liquid PPDA medium: 200 g of potato extract, 20 g of glucose, 10 g of peptone, 1.5 g of KH_2_PO_4_, 0.5 g of MgSO_4_, 20 mg of vitamin B_1_, and 1000 mL of distilled water; solid PPDA medium: 15% agar in liquid PPDA medium) at 13°C according to the previous method (Wu et al., 2020). In brief, the fungal colonies on the PPDA plates at 13°C for 60 days were transferred to 250-mL flasks containing 150 mL of liquid PM medium (200 g of potato extract, 20 g of maltose, 10 g of peptone, 1.5 g of KH_2_PO_4_, 0.5 g of MgSO_4_, 20 mg of vitamin B_1_, and 1000 mL of distilled water) (Liu et al., 2019). The flasks were incubated at 13°C on a 120-rpm rotary shaker, and, after 50 days, the blastospores from the flasks were collected by using three layers of sterile lens papers to remove hyphae and large particles, and, then, the filtered solution was centrifuged at 8,000 rpm for 15 min at 10°C and the supernatant discarded. Harvested blastospores were re-suspended in sterile phosphate-buffered saline (PBS; pH 6.2) at a concentration of 3.0 × 10^6^ blastospores per mL. The blastospore suspension was kept at 4°C for less than 3 days before use for larval challenge. The fungal isolate was identified by using the amplified sequence from the internal transcribed spacer (ITS; ITS1-5.8S-ITS2) of the nuclear ribosomal DNA as described by (Cao et al., 2015).

### Larval infection

Each fifth instar larva received 4 µL of suspension containing 1.2 × 10^4^ blastospores by a microinjection system (IM-31; Narishige, Japan). One hundred fifty larvae were employed for each replicate, and three replicates were established for each injection. Larvae injected with PBS buffer (pH 6.2) or without any injection were set as controls. The injected larvae were reared with *P*. *anserina* as food at 4°C for 1 week and then transferred to a culture room at 13°C. After 90 days, about 10 µL of hemolymph of each injected larva was sampled for confirming the presence of the blastospores under a microscopy (AXIO; Zeiss, Germany).

### RNA-seq

#### Preparation of RNA, library construction, and sequencing

For RNA-seq, the hemolymph (100 µL from each larva) was sampled, using micro-needles (pulled glass capillaries G-1 by a micropipette PC-10 puller, Narishige, Japan), from the living larvae uninjected (CK), with low blastospores load (BL, less than 20 blastospores per 10 μL of hemolymph) at 60 days after infection, and with high blastospores load (BH, more than 200 blastospores per 10 μL of hemolymph) at 180 days after infection. All these samples were treated in liquid nitrogen for at least 30 min and kept in -80°C for sequencing.

Total RNA of the samples was extracted with TRIzol according to the manufacturer’s instructions. A Nanodrop ND-2000 spectrophotometer, non-denaturing agarose gel electrophoresis, Qubit 2.0, and Agilent 2100 Bioanalyzer (Agilent, Santa Clara, CA, USA) were used to determine the quantity and quality of RNA in the three samples. A total of 15 individual cDNA libraries were constructed from blastospore samples (BL and BH) and control sample (CK). Three biological replicates for each sample were established, and 10 larvae were used for each biological replicate. The quantification and qualification of the libraries were analyzed on Qubit 2.0, Agilent 2100 Bioanalyzer (Agilent, Santa Clara, CA, USA), and ABI StepOnePlus Real-Time PCR system (ABI, Waltham, MA, USA). An Illumina NovaSeq 6000 platform (Illumina, San Diego, CA, USA) was used for sequencing. All raw sequence data were deposited in the National Center for Biotechnology Information (NCBI) Sequence Read Archive under the BioProject accession number: SUB14404655.

#### Mapping, annotation, and differentially expressed gene analysis

The sequenced reads containing adapter sequences with more than 10% uncertain base pairs and low quality were removed. The resulting clean reads were used to evaluate the quality through base composition and quality distribution. Only the clean reads with a balanced composition, as well as high distribution of high-quality base (sequencing quality value > 20) were kept. The remaining clean reads were mapped to the genome of *O*. *sinensis* and *T*. *xiaojinensis* at the same time using HISAT2 (2.0.6) (Trapnell et al., 2009), and the clean reads that finally mapped to *O*. *sinensis* genome were kept. StringTie (v1.0.4) was used to reconstruct transcript (Pertea et al., 2015), and the potential novel transcripts were predicted by cufflinks (v2.2.1) (Trapnell et al., 2012). All novel transcripts were annotated against the NCBI non-redundant protein database and Swiss-Prot database using BlastX (E-value < 1e−5). RSEM (v 1.2.31, RNA-seq by expectation maximization) (Li and Dewey, 2011), a utility package in the software Trinity, was used to estimate the abundance of transcripts and the fragments per kilobase per million mapped read (FPKM) for the digital gene expression profile. DEGs were calculated using edgeR (Robinson et al., 2010). *P*-values were corrected for multiple hypothesis tests, and the threshold *p*-value by false discovery rate (FDR) was determined. Genes in different samples with FDR < 0.05 and |log2foldchange| > 1 were considered as DEGs. The expression pattern of DEGs across three samples were clustered by using hierarchical clustering performed in R package (Rao et al., 2019a).

#### Functional enrichment analysis

The Gene Ontology (GO) enrichment analysis was performed with the Database for Annotation, Visualization, and Integrated Discovery (DAVID v6.8) (Dennis et al., 2003). GO visualization was performed by TopGO (v2.40.0) package from R software (Alexa and Rahnenführer, 2020). Pathway enrichment analysis was performed by Kyoto Encyclopedia of Genes and Genomes (KEGG) Orthology-Based Annotation System (v3.0) (Xie et al., 2011) with a threshold *p*-value ≤ 0.05. All the expressed genes were also analyzed by Gene Set Enrichment Analysis (v4.1.0) (Subramanian et al., 2005, 2007). The GO and KEGG annotation of all genes in *T*. *xiaojinensis* were used as gene set database. The gene sets with FDR *q*-value < 0.05 were considered statistically significant.

### Knockdown of key DEGs in *T*. *xiaojinensis* larvae

Eight genes (**Supplementary Table 1**) upregulated in the larvae containing high blastospore load were selected for knockdown by RNAi. M5 HiPer T7 *In Vitro* Transcription T7 was used to synthesize siRNAs of the selected genes according to the product instruction. If the gene has more copies, then the si*RNA* of all copies, including those not upregulated by high blastospore load, were synthesized and mixed for use. The primers were designed on the basis of the instruction of the website software (http://rnaidesigner.thermofisher.com/rnaiexpress/design.do) (**Supplementary Table 2**) and synthesized by Sangon (Shanghai China). The sixth instar *T*. *xiaojinensis* larvae with almost pure blastospores in the hemolymph at 140 days after injection were anesthetized with nitrogen, and si*RNAs* (4,000 ng/μL) in 4 μL of ribonuclease (RNase)-free water were injected into each larva using a microinjector (Narishige, Tokyo, Japan) at 16°C. si Green Fluorescent Protein (*GFP*) and RNA-free water–injected control groups were included. Ten larvae for each replicate with three replicates were used for each gene. The injected larvae were placed into the wells of cell plates without food and kept at 12°C–14°C. After 5 days, the rates of larval mortality, mummification, ecdysis, and pupation were recorded, and the blastospores and prehyphae in the hemolymph were observed and estimated under a fluorescence microscopy, after stained with Calcofluor White (Sigma), a vital stain that binds to β-glucans (Xie et al., 2022), and diluted with sterile PBS (pH 6.2). The whole experiment was repeated twice.

### Quantitative real-time PCR of knockdown key genes

The extracted RNA from the gut-free *Thitarodes* larvae receiving siRNAs was used to verify the RNAi functions. A total of 1 μg of RNA from each treatment was used for cDNA synthesis according to the manufacturer’s protocol (ToloScript All-in-one RT EasyMix for qPCR). The 25-μL reaction consisted of 1 μL of diluted cDNA (1:2), 10 μL of 2×TransStart Tip Green qPCR SuperMix, and 10 µM of each primer in 0.4 µL were used for the quantitative real-time (qRT)–PCR reaction. According to the manufacturer’s instructions, all reactions were performed on a CFX96 Connect™ Real-Time System (Bio-Rad, USA). Thermal cycling conditions were set to 95°C for 30 s of initial denaturation, followed by 40 cycles of 95°C for 10 s, 55°C for 15 s, and 72°C for 10 s of amplification. Then, a melting curve analysis from 65°C to 95°C was applied to all reactions to ensure consistency and specificity of the amplified product. Primers were designed by NCBI and then blasted search with *T*. *xiaojinensis* genome to confirm the specificity. All primers used for the testing genes were described in **Supplementary Table 3**. Quantitative measurements were normalized by the reference gene *Translation elongation factor 2* (*EF2*), and relative expression levels were calculated using 2^−ΔΔCt^ method (Livak and Schmittgen, 2001; Vandesompele et al., 2002). In the regression analysis, the fold changes of RNA-seq were base-2 logarithm of FPKM ratios, and the fold changes of qRT-PCR were ΔΔCt (Livak and Schmittgen, 2001).

## Results

### RNA sequencing analysis

**Figure 1:**
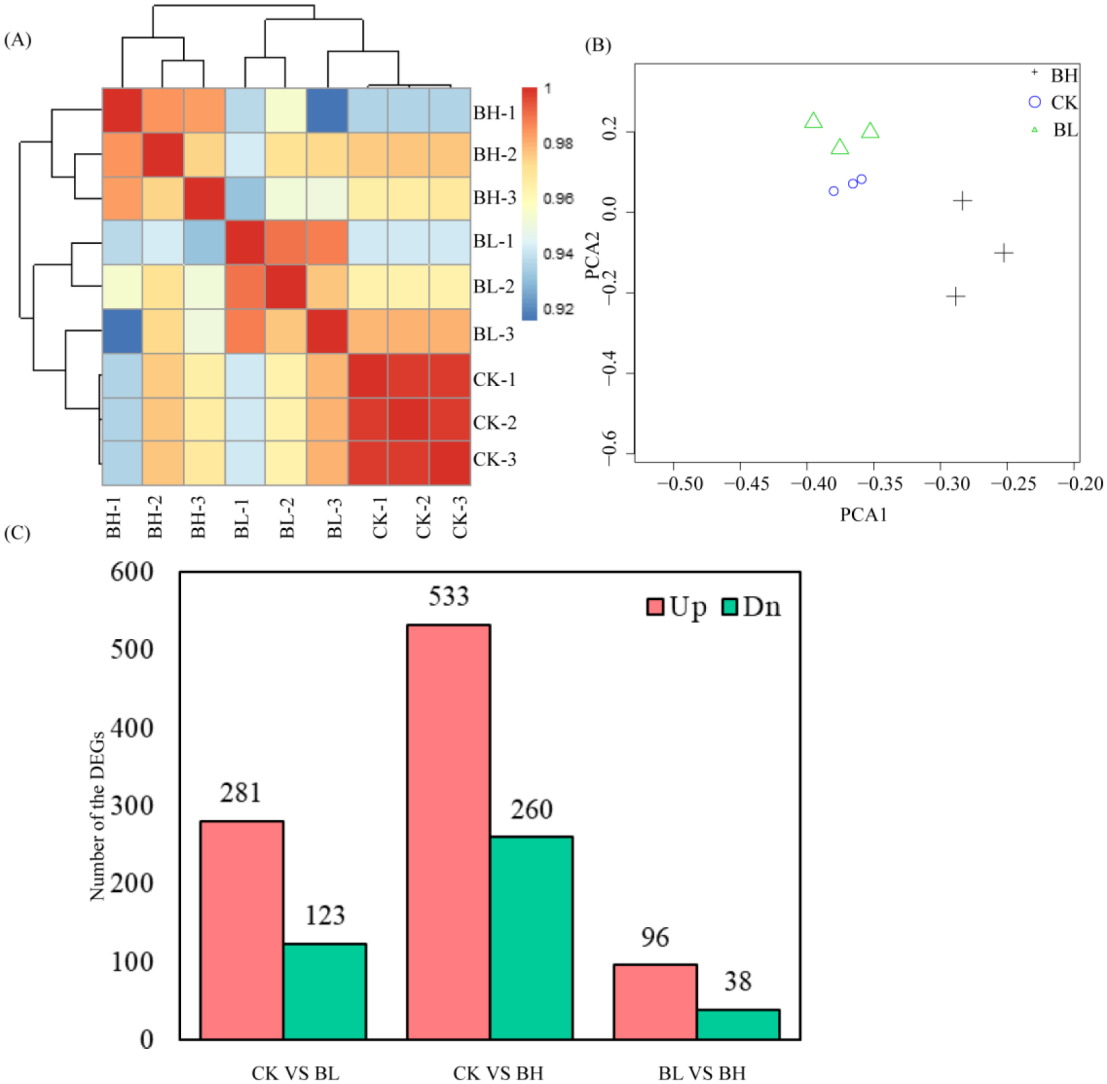
Differentially expressed genes analysis. **(A)** Heatmap of duplicate samples of different larval stages of *Thitarodes xiaojinensis* with different concentrations of *O. sinensis* blastospores. The color spectrum, ranging from blue through yellow to red, represents Pearson correlation coefficients ranging from 0.92? to 1, indicating low to high correlations. **(B)** Principal component analysis of the transcriptome for different larval stages of *T. xiaojinensis* with different concentrations of *O. sinensis* blastospores. **(C)** Unique and common DEGs for different comparison groups.

Whole-genome mRNA sequencing was employed to determine the gene expressions in the hemolymph of *T*. *xiaojinensis* larvae containing different concentrations of *O*. *sinensis* blastospores. In total, 86.33 Gb of high-quality clean data was obtained from *T*. *xiaojinensis* (**Supplementary Table 4**). A total of nine libraries of three larval stages were constructed, including CK (without *O*. *sinensis* fungus), BL, BH, with three biological replicates for each stage. At least 90.04% of clean reads match the corresponding genome using HISAT2 (**Supplementary Table 4**). The correlation coefficient between the biologically replicated samples was shown in **Figure 1**. The correlation coefficient between the biological duplicates in the same stage exceeded 0.92, which is always higher than that between different larval stages (**Figure 1A**). Similar results were obtained in the principal component analysis (**Figure 1B**). The FPKM value of expressed genes in nine samples was shown in **Supplementary Table 5**.

To gain insights into global transcriptional changes in *T*. *xiaojinensis* in different stages (BL and BH, together with CK), pairwise comparisons were performed between the consecutive developmental stages. A total of 840 genes were differentially expressed across all developmental stages (|log2foldchange| > 1 and FDR < 0.05), and 2,928 of which were differentially expressed between consecutive stages, including 404 (281 upregulated and 123 downregulated), 793 (533 upregulated and 260 downregulated), and 134 (96 upregulated and 38 downregulated) DEGs from the compared groups CK vs. BL, CK vs. BH, and BL vs. BH, respectively (**Supplementary Table 1**; **Figure 1C**).

From two blastospore stages (BL vs. BH), 96 genes of the total 134 DEGs were upregulated at BH stage, and 11 upregulated genes were not expressed at BL stage (**Supplementary Table 1**). The top 20 up-expressed genes by log2foldchange included *fibroin light chain*; *cuticle protein 18.6*, *isoform B*; uncharacterized gene 1; *lipase member H*; *XP_026486906.1 cuticle protein 18.7-like* (*Vanessa tameamea*); uncharacterized gene 2; *larval/pupal rigid cuticle protein 66*; uncharacterized gene 3; *pupal cuticle protein Edg-84A*; uncharacterized gene 4; *AAV91426.1 putative protease inhibitor 4* (*Lonomia obliqua*); *pupal cuticle protein Edg-78E*; *Acyl-CoA Delta(11) desaturase*; *XP_026486906.1 cuticle protein 18.7-like* (*Vanessa tameamea*); *lactase-phlorizin hydrolase*; and *serine protease inhibitor 3/4* (Fragment). Nine of the 20 top upregulated genes were cuticles proteins (**Supplementary Table 1**), indicating that cuticle proteins in *T*. *xiaojinensis* highly activated when the blastospores were enriched in the larval hemolymph.

On the other hand, 5 of the 38 downregulated genes were not expressed at BH stage. The top 10 downregulated genes include almost all uncharacterized proteins, such as XP_026731970.1 uncharacterized protein LOC113496800 (*Trichoplusia ni*), uncharacterized protein, XP_026327351.1 glycine-rich cell wall structural protein (*Hyposmocoma kahamanoa*), XP_026754401.1 uncharacterized protein LOC113514510 (Galleria mellonella), XP_026492458.1 uncharacterized protein LOC113398093 (*Vanessa tameamea*), XP_026759163.1 eisosome protein SEG2 (*Galleria mellonella*), XP_026734645.1 uncharacterized protein LOC113498716 (*Trichoplusia ni*), XP_026492446.1 uncharacterized protein LOC113398080 (*Vanessa tameamea*), Lipase member I, and XP_008195762.1 PREDICTED: uncharacterized protein LOC103313673 (*Tribolium castaneum*).

**Figure 2:**
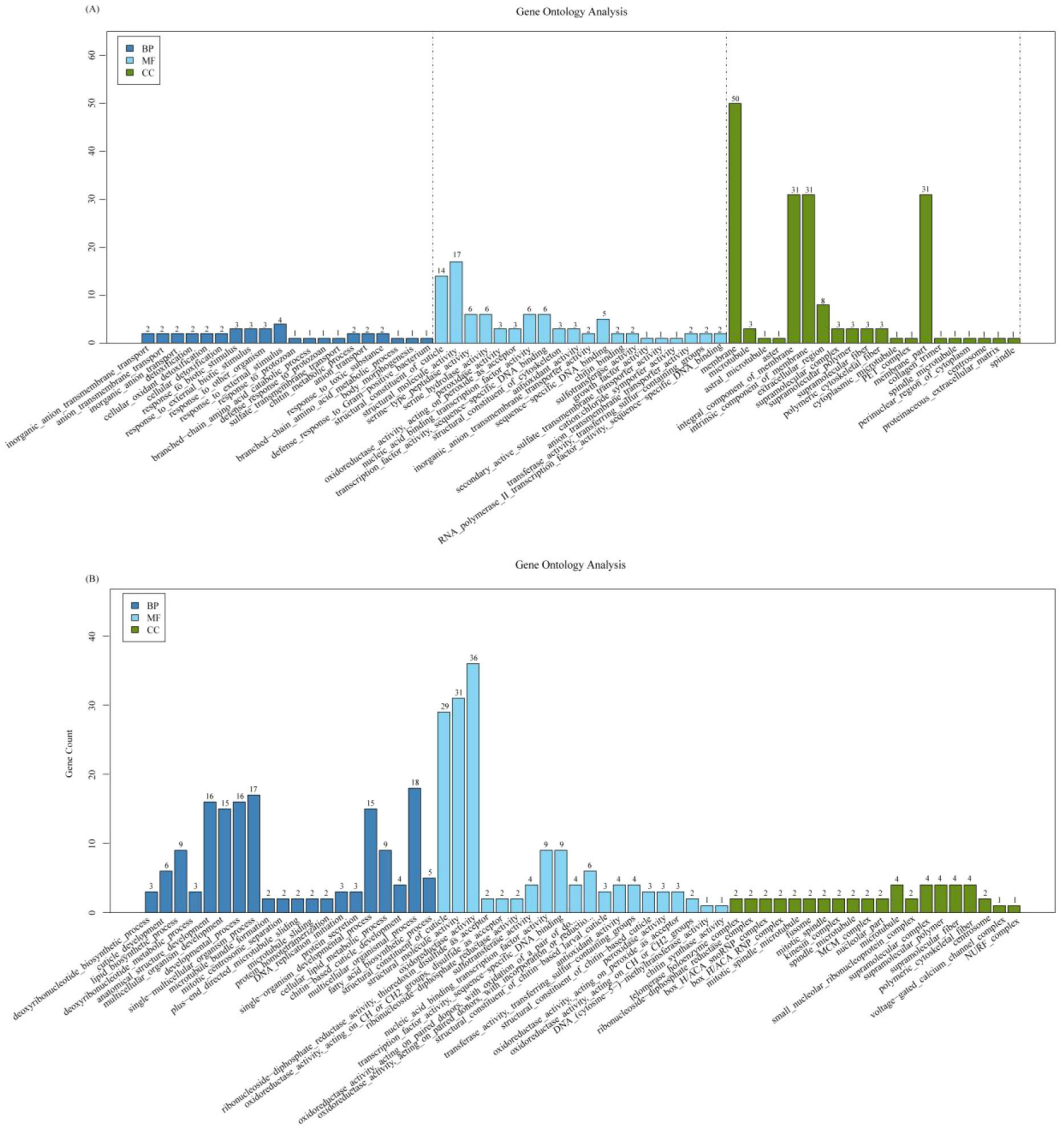
GO enrichment analysis. **(A)** GO enrichment analysis of the upregulated gene between high blastospore load (BH) 180 days after infection and non-injected live larvae (CK). BH epidermal structure–related genes were significantly enriched. **(B)** GO enrichment analysis of the upregulated gene between low blastospore load (BL) at 60 days after infection and non-injected live larvae (CK). BL membrane structure–related genes were significantly enriched. BP, biological process; MF, molecular function; CC, cellular component.

GO analysis indicated that sugar metabolism, degradation of secondary compounds, coenzyme, and vitamin metabolism was activated in the larvae after *O*. *sinensis* infection for 140 days (**Figure 2**). The pathways for insect hormone biosynthesis and cell cycle were significantly enhanced in BH; however, tyrosine metabolism pathway was downregulated in both BL and BH stages (**Figure 2**).

**Figure 3:**
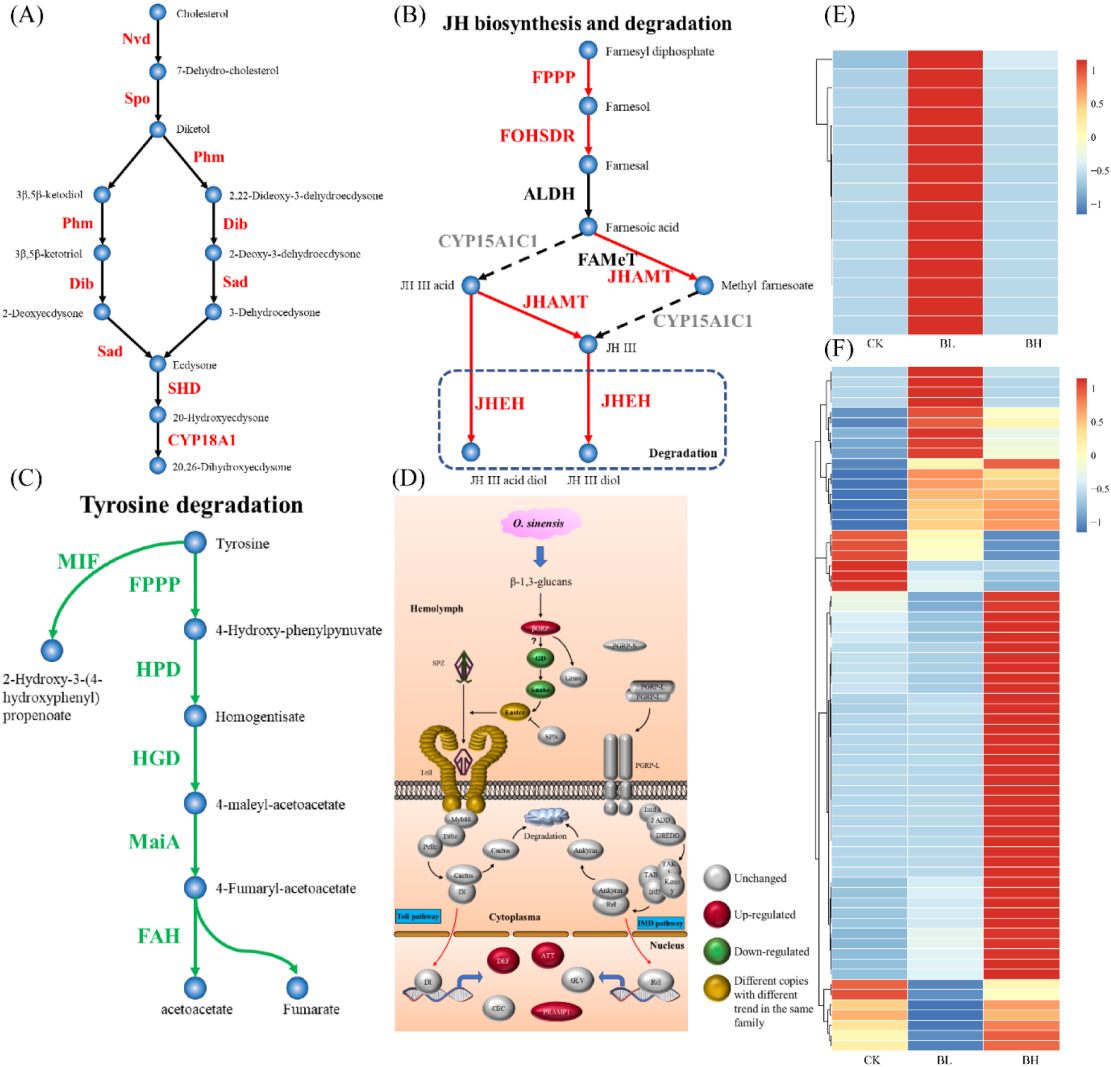
Analysis of important pathways. **(A)** Ecdysone synthesis pathway (Belles et al., 2005). **(B)** Juvenile hormone synthesis pathway (Belles et al., 2005). **(C)** Fumaric acid and acetoacetic acid pathways (Boyce et al., 2015). **(D)** Humoral immune pathway (Rao et al., 2019a). **(E)** Expression of membrane structure–related genes. **(F)** Expression of cuticle protein-related genes. The color spectrum, ranging from blue through yellow to red, indicates that the gene expression level is from −1 to 1.

Important pathways for the synthesis of 20E and juvenile hormone (JH), tyrosine degradation, dopa metabolism, trehalase metabolism, UDP-glucuronosyltransferase (UGT) function, humoral immunity, membrane structure formation, and cuticle proteins were analyzed (**Figure 3**; **Supplementary Table 6**). Related genes (*Nvd*, *Spo*, *Phm*, *Dib*, *Sad*, and *Shd*) for synthesis of 20E were regulated, and CYP18A1, an enzyme transforming 20E to 20,26-dihydroxyecdysone, was also enriched (**Figure 3A**), indicating the activation of this pathway in the larvae after fungal infection. For JH synthesis, the key gene *CYP15A1C1* was not active, although other genes (*FPPP*, *FOHSDR*, and *JHMAT*) were upregulated and *JHEHT* for degradation of JH was active (**Figure 3B**), implying that the JH synthesis pathway appeared not to be induced by the fungal infection. The genes (*FPPP*, *HPD*, *HGD*, *MaiA*, and *FAH*) for tyrosine degradation were suppressed, which might inhibit the formation of acetoacetate and fumarate (**Figure 3C**). Aromatic-L-amino-acid/L-tryptophan decarboxylase were downregulated, indicating that the pathway from L-dopa to dopamine was suppressed. Trehalase (*TREH*) was upregulated, implying the active metabolism of trehalose (which is dominant sugar in insect hemolymph) to D-glucose. Most of the genes involved in the immune response were unchanged when the hemolymph was full of blastospores, and only several genes for the following proteins (Beta-1,3-glucan-binding protein for recognition receptor; protein toll, protein spaetzle, and serine protease easter for Toll pathway; and attacins, PRAMP1-1, and defensin-B for immune response) were enhanced (**Figure 3D**). It seemed that membrane structure formation was active when the larvae were challenged by the fungus, as the related genes were highly upregulated (**Figure 3E**). Especially, after fungal infection, 67 genes encoding cuticle proteins were differentially expressed (**Figure 3F**), accounting for 39% of the total cuticle proteins in *T*. *xiaojinensis*.

### Dimorphic transition of *O*. *sinensis* conferred by larval genes

The rates of mortality, ecdysis, and mummification of the larvae containing high load of blastospores were not significantly different after receiving siRNAs of all eight genes (data not shown).

As indicated in **Table 1**, the living larvae that received siRNAs of the following genes *Pupal cuticle protein*, *ecdysone-induced protein 78C*, and *multidrug resistance protein 1*, together with green fluorescent protein (GFP) and H_2_O, after 120 h, contained pure blastospores in the hemolymph, indicating that knockdown of these larval genes did not influence the fungal transition. Interestingly, the living larvae with different percentages were found to harbor prehyphae in the hemolymph, after challenged with siRNAs of the genes *Flightin* (83.33 ± 11.02), *larval cuticle protein LCP-30* (78.25 ± 3.55%), *26-hydroxylase* (*CYP18A1*) (40.48 ± 6.30%), *cuticle protein 18*.*6, isoform B* (39.81 ± 2.31%), and *probable chitinase 3* (34.52 ± 7.81%). It appeared that more living larvae treated by siRNAs of *flightin*, *larval cuticle protein LCP-30*, or *larval/pupal rigid cuticle protein 66* harbored prehyphae in the hemolymph than those treated by siRNAs of *26-hydroxylase* (*CYP18A1*), *cuticle protein 18*.*6, isoform B*, or *probable chitinase 3*. The larvae receiving siRNA of *probable chitinase 3* harbored more blastospores than those larvae with siRNAs of *flightin*, *26-hydroxylase* (*CYP18A1*), *larval cuticle protein LCP-30*, *multidrug resistance protein 1*, and *cuticle protein 18*.*6, isoform B*, together with the larvae from the control. The prehyphae per larva also varied with the knockdown genes, from 2.58 ± 0.51 × 10^8^/mL to 7.65 ± 0.91 × 10^8^/mL (**Table 1**). All these results indicated that the knockdown of the seven larval genes such as *flightin*, *larval cuticle protein LCP-30*, *26-hydroxylase* (*CYP18A1*), *cuticle protein 18*.*6, isoform B*, and *probable chitinase 3* significantly stimulated the dimorphic transition from blastospores to prehyphae in *O*. *sinensis* fungus in the larval hemolymph.

**Table 1:** Influence of larval gene knockdown on the fungal dimorphic transition after 120 hours post injection.

Genes	Living larvae containing blastospores (%)	Living larvae containing prehyphae (%)	Blastospores per larva(x10^8^/mL)	Prehyphae per larva(x10^8^/mL)
*26-hydroxylase* (*CYP18A1*)	59.52±6.3 b	40.48±6.30 b	8.97±0.88 b	6.04±1.33 ab
*Cuticle protein 18.6, isoform B*	60.19±2.31 b	39.81±2.31 b	10.18±0.78 b	2.58±0.51 b
*Ecdysone-induced protein 78C*	100 a		12.83±1.26 ab	
*Flightin*	16.67±11.02 c	83.33±11.02 a	8.83±1.06 b	6.39±0.63 a
*Larval cuticle protein LCP-30*	21.75±3.55 c	78.25±3.55 a	9.87±0.85 b	3.89±0.53 ab
*Multidrug resistance protein 1*	100 a		9.54±0.44 b	
*Probable chitinase 3*	65.48±7.81 b	34.52±7.81 b	14.43±0.72 a	4.06±0.47 ab
*Pupal cuticle protein *	100 a		12.8±1.12 ab	
*GFP*	100 a		10.34±0.68 ab	
H_2_O	100 a		10.45±0.63 ab	

The data was expressed as mean±SEM at 120 hours post larval injection. The columns with different letters indicated significant differences (Tukey test, p<0.05). The larval genes were knockdown by injecting the corresponding siRNA (4000 ng/μL of siRNAs in 4 μL of RNase-free water) into the haemolymph of infected larvae full of blastospores only.

### Verification of gene knockdown by qRT-PCR

**Figure 4:**
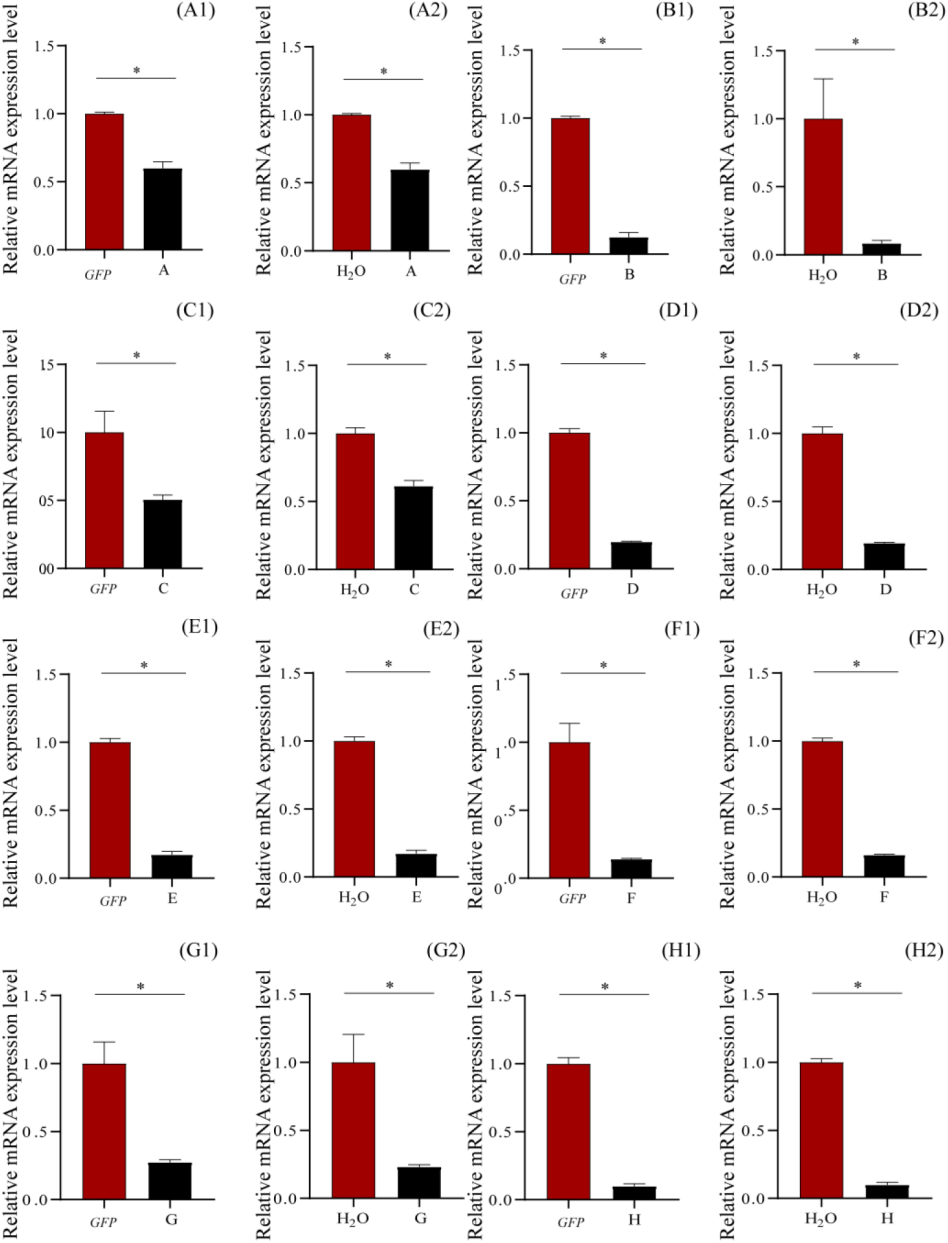
Relative mRNA expression levels of the knockdown genes by qRT-PCR after 120 hours post injection. **(A)** *Pupal cuticle protein*; **(B)** *Ecdysone-induced protein* 78C; **(C)** Flightin; **(D)** *26-hydroxylase*; **(E)** *Larval cuticle protein* LCP-30; **(F)** *Multidrug resistance protein 1*; **(G)** *Probable chitinase* 3; **(H)** *Cuticle protein 18.6, isoform B*. 1, injection with dsGFP; 2, injection with H_2_O.

RNAi knockdown of *ecdysone-induced protein 78C* and *flightin* in one copy, *pupal cuticle protein* and *26-hydroxylase* in two copies, *multidrug resistance protein 1* and *probable chitinase 3* in four copies, *larval cuticle protein LCP-30* in five copies, and *cuticle protein 18*.*6* in seven copies suppressed the expressions of these genes in various levels (**Figure 4**), compared to those of GFP and H_2_O, being 87.43% and 91.54% (compared to GFP and H_2_O, respectively) for *ecdysone-induced protein 78C*, 49.44% and 38.64% for *Flightin*, 40.06% and 40.14% for *pupal cuticle protein*, 80.18% and 80.64% for *26-hydroxylase*, 85.92% and 83.84% for *multidrug resistance protein 1*, 72.56% and 76.81% for *probable chitinase 3*, 82.73% and 82.81% for *larval cuticle protein LCP-30*, and 90.05% and 90.12% for *cuticle protein 18*.*6* (**Figure 4**). These results demonstrated that injection of the siRNAs of these genes into the larval hemolymph caused the gene silence in *T*. *xiaojinensis* larvae.

## Discussion

Commercial cultivation of Chinese cordyceps has been successfully established to meet the market demand (Li et al., 2016; Han et al., 2019; Li et al., 2019; Liu et al., 2020). However, low and slow mummification rate seriously constrained the efficient production of Chinese cordyceps (Zhou et al., 2014; Lu et al., 2015; Qin et al., 2018). Although the high larval microbiological diversities influenced by *O*. *sinensis* challenge and the involvement of dominant bacteria during the mummification process of infected larvae are discovered (Wu et al., 2020), how the host larvae influence their parasitic fungus in the hemolymph becomes unsolved questions. In this study, it is demonstrated, for the first time, that siRNA knockdown of some selected larval genes in the hemolymph of *T*. *xiaojinensis* host significantly causes the dimorphic transition from blastospores to prehyphae of *O*. *sinensis* fungus. Especially, 9 of the 20 top upregulated genes encoding cuticle proteins at BH stage are involved in this process. These results indicate that enriched blastospores highly activated the expression of cuticle proteins in *T*. *xiaojinensis* in the larval hemolymph, which plays a role in maintaining blastospore stage, providing novel insights into explaining this unique parasitic interaction of *T*. *xiaojinensis* and *O*. *sinensis* fungus.

Cuticle proteins are parts of insect cuticles that act as barriers for insect pathogens and play important roles in insect development, propagation, resistance to insecticides, and anti-fungi. So far, 13 cuticle protein families (CPR, CPF, CPFL, CPT, CPG, CPAPs, CPH, and CPLC) have been reported, with biggest CPR family (RR-1, RR-2, and RR-3 subfamilies), which contains R&R Consensus with Chitin-binding domain 4 (CBD4) (Zhou et al., 2016; Pan et al., 2018). Mutation of a member of an unconventional cuticle protein family Tweedle D alters body shape in *Drosophila* (Guan et al., 2006). Wing-specific cuticular protein Lm ACP7 is essential for normal wing morphogenesis in the migratory locust (Zhao et al., 2019). Knockdown of specific cuticular proteins analogous to *peritrophin 3* gene disrupts larval and ovarian development in *Bactrocera dorsalis* (Hou et al., 2021). Cuticle proteins are regarded as critical determinants in insecticide resistance (Balabanidou et al., 2018), such as pyrethroid and deltamethrin resistance in *Culex pipiens pallens* (Fang et al., 2015; Huang et al., 2018; Xu et al., 2020). Some cuticular proteins in *Tribolium castaneum* show antifungal roles (Sirasoonthorn et al., 2021). Cuticle proteins are involved in insect immunity. Overexpression of BmCPT1 cuticle protein in silkworm causes upregulation of *BmRelish1* and *gloverin*, indicating the involvement of BmCPT1 in insect immunity (Li et al., 2015). Cuticular protein genes are upregulated in silkworm larvae by *Beauveria bassiana* (Xing et al., 2017) and *Cordyceps militaris* (Kato et al., 2022) conidia injection, suggesting that the insects respond to the invasive fungi by rearrange the composition of cuticular proteins. Cuticle proteins in *Hepialus altaicola* larvae are also upregulated under cold stress (Sun et al., 2021). Insect cuticle proteins in different developmental stages and tissues are also regulated by insect pheromones (Charles, 2010). Wing-specific cuticular protein BmWCP1-9 in silkworms by 20E signal pathways (Deng et al., 2011). Nuclear receptor *LmHR3* induced by 20E controls locust molting by regulating chitin synthesis and degradation in *Locusta migratoria* (Zhao et al., 2018). Cuticle protein *LmNCP4*.*9* in the fifth-instar locust nymphs was upregulated by different concentrations of 20E but downregulated by juvenile hormone analog (Zhao et al., 2021). As described above, insect cuticle proteins are involved in insect immunity (Li et al., 2015) and regulated by insect pheromones (Charles, 2010; Deng et al., 2011; Zhao et al., 2018, 2021). In this study, for the first time, it is demonstrated that the fungal transition can be regulated by lowering the expression of cuticle proteins of *Thitarodes* larvae. However, whether the cuticle proteins directly influence the fungal transition or regulate the blastospores via other genes is unknown.

High percentage (83.33 ± 11.02%) of the living larvae harbored prehyphae in the hemolymph, after challenged with siRNAs of the gene *flightin*. *Flightin* is a 20-kDa myofibrillar protein first identified in *Drosophila* indirect flight muscle, and contributes to the proper assembly of thick filaments and be required for the integrity of thick filaments and sarcomere (Reedy et al., 2001; Vigoreaux, 2006; Contompasis et al., 2010; Soto Adames et al., 2013). In addition to having a known function in insect flight and locomotion (Ayer and Vigoreaux, 2003), *flightin* also drives wing movement as well as vibration of the male-specific tymbal (Xue et al., 2013; Chen et al., 2019) and shows an association between *flightin* genetic variation and cold tolerance or thermal adaptation in general (De Jong and Saastamoinen, 2018). To the best of our knowledge, this study is the first to demonstrate the across regulation of fungal dimorphism by host *flightin* gene.

## Conclusion

A unique parasitic complex of *Thitarodes*/*Hepialus* ghost moths and *Ophiocordyceps sinensis* fungus in the Tibetan Plateau provides an excellent model for studying insect-fungus interaction. Key larval genes in *T*. *xiaojinensis* critical for *O*. *sinensis* blastospore development or filamentation were identified by RNA-seq. Nine of the 20 top upregulated genes encoded cuticles proteins when the larval hemolymph was full of blastospores, indicating the active involvement of these proteins in regulation of the invasive fungus. siRNA knockdown of five larval genes (*cuticle protein 18.6*, *Flightin*, *26-hydroxylase*, *larval cuticle protein LCP-30*, and *probable chitinase 3*) in the hemolymph of *T*. *xiaojinensis* larvae with high load of blastospores significantly stimulated the dimorphic transition from blastospores to prehyphae of *O*. *sinensis* fungus. The expressions of these genes determined by qRT-PCR were suppressed. All these results provide novel knowledge on the regulation of *O*. *sinensis* fungal dimorphism by *Thitarodes* host and cues for further study of *Thitarodes* biology and commercial cultivation of Chinese cordyceps.

## Data Availability

The datasets presented in this study can be found in online repositories. The names of the repository/repositories and accession number(s) can be found in the article/**Supplementary Material**.
