# Supplementary material for: Knockdown of Thitarodes host genes influences dimorphic transition of Ophiocordyceps sinensis in the host hemolymph
Source: Front Cell Infect Microbiol. 2024 Sep 27;14:1451628. doi: 10.3389/fcimb.2024.1451628 (PMC11466941; doi:10.3389/fcimb.2024.1451628)
Supplement: Supplementary file 2 [file Table2.docx]

**TABLE S2 |** Primers used for siRNA synthesis

| Name | Sequence5’-3’ |
| --- | --- |
| *26*-*hydroxylase* (*CYP18A1*)- A1 | TAATACGACTCACTATAGGG **GCAGTTCAATCGAAATGTC** |
| *26*-*hydroxylase* (*CYP18A1*)- A2 | **GACATTTCGATTGAACTGC** CCCTATAGTGAGTCGTATTA |
| *26*-*hydroxylase* (*CYP18A1*)- A3 | TAATACGACTCACTATAGGG **GACATTTCGATTGAACTGC** |
| *26*-*hydroxylase* (*CYP18A1*)- A4 | **GCAGTTCAATCGAAATGTC** CCCTATAGTGAGTCGTATTA |
| *26*-*hydroxylase* (*CYP18A1*)- B1 | TAATACGACTCACTATAGGG **GCTACTTGCACTCTGTGTC** |
| *26*-*hydroxylase* (*CYP18A1*)- B2 | **GACACAGAGTGCAAGTAGC** CCCTATAGTGAGTCGTATTA |
| *26*-*hydroxylase* (*CYP18A1*)- B3 | TAATACGACTCACTATAGGG **GACACAGAGTGCAAGTAGC** |
| *26*-*hydroxylase* (*CYP18A1*)- B4 | **GCTACTTGCACTCTGTGTC** CCCTATAGTGAGTCGTATTA |
| *Cuticle protein 18*.*6*, *isoform B*-A1 | TAATACGACTCACTATAGGG **GGAAATTCACCATCATCCC** |
| *Cuticle protein 18*.*6*, *isoform B*-A2 | **GGGATGATGGTGAATTTCC** CCCTATAGTGAGTCGTATTA |
| *Cuticle protein 18*.*6*, *isoform B*-A3 | TAATACGACTCACTATAGGG **GGGATGATGGTGAATTTCC** |
| *Cuticle protein 18*.*6*, *isoform B*-A4 | **GGAAATTCACCATCATCCC** CCCTATAGTGAGTCGTATTA |
| *Cuticle protein 18*.*6*, *isoform B*-B1 | TAATACGACTCACTATAGGG **GTTGAGGAAATTCACCATC** |
| *Cuticle protein 18*.*6*, *isoform B*-B2 | **GATGGTGAATTTCCTCAAC** CCCTATAGTGAGTCGTATTA |
| *Cuticle protein 18*.*6*, *isoform B*-B3 | TAATACGACTCACTATAGGG **GATGGTGAATTTCCTCAAC** |
| *Cuticle protein 18*.*6*, *isoform B*-B4 | **GTTGAGGAAATTCACCATC** CCCTATAGTGAGTCGTATTA |
| *Cuticle protein 18*.*6*, *isoform B*-C1 | TAATACGACTCACTATAGGG **GCCGGATACGGACAATTTC** |
| *Cuticle protein 18*.*6*, *isoform B*-C2 | **GAAATTGTCCGTATCCGGC** CCCTATAGTGAGTCGTATTA |
| *Cuticle protein 18*.*6*, *isoform B*-C3 | TAATACGACTCACTATAGGG **GAAATTGTCCGTATCCGGC** |
| *Cuticle protein 18*.*6*, *isoform B*-C4 | **GCCGGATACGGACAATTTC** CCCTATAGTGAGTCGTATTA |
| *Ecdysone*-*induced protein 78C* (*E78C*)-1 | TAATACGACTCACTATAGGG **GACACGCTACAGAGTCCAC** |
| *Ecdysone*-*induced protein 78C* (*E78C*)-2 | **GTGGACTCTGTAGCGTGTC** CCCTATAGTGAGTCGTATTA |
| *Ecdysone*-*induced protein 78C* (*E78C*)-3 | TAATACGACTCACTATAGGG **GTGGACTCTGTAGCGTGTC** |
| *Ecdysone*-*induced protein 78C* (*E78C*)-4 | **GACACGCTACAGAGTCCAC** CCCTATAGTGAGTCGTATTA |
| *Flightin*-1 | TAATACGACTCACTATAGGG **GCAAATGATGTGAAACCTC** |
| *Flightin*-2 | **GAGGTTTCACATCATTTGC** CCCTATAGTGAGTCGTATTA |
| *Flightin*-3 | TAATACGACTCACTATAGGG **GAGGTTTCACATCATTTGC** |
| *Flightin*-4 | **GCAAATGATGTGAAACCTC** CCCTATAGTGAGTCGTATTA |
| *Larval cuticle protein LCP*-*30*-A1 | TAATACGACTCACTATAGGG **GACAATGAGCGTGAAGGTC** |
| *Larval cuticle protein LCP*-*30*-A2 | **GACCTTCACGCTCATTGTC** CCCTATAGTGAGTCGTATTA |
| *Larval cuticle protein LCP*-*30*-A3 | TAATACGACTCACTATAGGG **GACCTTCACGCTCATTGTC** |
| *Larval cuticle protein LCP*-*30*-A4 | **GACAATGAGCGTGAAGGTC** CCCTATAGTGAGTCGTATTA |
| *Larval cuticle protein LCP*-*30*-B1 | TAATACGACTCACTATAGGG **GAGCCACCAAAGGACATTC** |
| *Larval cuticle protein LCP*-*30*-B2 | **GAATGTCCTTTGGTGGCTC** CCCTATAGTGAGTCGTATTA |
| *Larval cuticle protein LCP*-*30*-B3 | TAATACGACTCACTATAGGG **GAATGTCCTTTGGTGGCTC** |
| *Larval cuticle protein LCP*-*30*-B4 | **GAGCCACCAAAGGACATTC** CCCTATAGTGAGTCGTATTA |
| *Larval cuticle protein LCP*-*30*-C1 | TAATACGACTCACTATAGGG **GCATACTGAAACAGGATAC** |
| *Larval cuticle protein LCP*-*30*-C2 | **GTATCCTGTTTCAGTATGC** CCCTATAGTGAGTCGTATTA |
| *Larval cuticle protein LCP*-*30*-C3 | TAATACGACTCACTATAGGG **GTATCCTGTTTCAGTATGC** |
| *Larval cuticle protein LCP*-*30*-C4 | **GCATACTGAAACAGGATAC** CCCTATAGTGAGTCGTATTA |
| *Larval cuticle protein LCP*-*30*-D1 | TAATACGACTCACTATAGGG **GAGGCCGTATTTATCAACC** |
| *Larval cuticle protein LCP*-*30*-D2 | **GGTTGATAAATACGGCCTC** CCCTATAGTGAGTCGTATTA |
| *Larval cuticle protein LCP*-*30*-D3 | TAATACGACTCACTATAGGG **GGTTGATAAATACGGCCTC** |
| *Larval cuticle protein LCP*-*30*-D4 | **GAGGCCGTATTTATCAACC** CCCTATAGTGAGTCGTATTA |
| *Larval cuticle protein LCP*-*30*-E1 | TAATACGACTCACTATAGGG **GTGGATTGATTACCAGTTC** |
| *Larval cuticle protein LCP*-*30*-E2 | **GAACTGGTAATCAATCCAC**CCCTATAGTGAGTCGTATTA |
| *Larval cuticle protein LCP*-*30*-E3 | TAATACGACTCACTATAGGG **GAACTGGTAATCAATCCAC** |
| *Larval cuticle protein LCP*-*30*-E4 | **GTGGATTGATTACCAGTTC** CCCTATAGTGAGTCGTATTA |
| *Multidrug resistance protein 1*-A1 | TAATACGACTCACTATAGGG **GGAATCCTAATGGCTGCAC** |
| *Multidrug resistance protein 1*-A2 | **GTGCAGCCATTAGGATTCC** CCCTATAGTGAGTCGTATTA |
| *Multidrug resistance protein 1*-A3 | TAATACGACTCACTATAGGG **GTGCAGCCATTAGGATTCC** |
| *Multidrug resistance protein 1*-A4 | **GGAATCCTAATGGCTGCAC**CCCTATAGTGAGTCGTATTA |
| *Multidrug resistance protein 1*-B1 | TAATACGACTCACTATAGGG **GCTCATGATAAGCGACTTC** |
| *Multidrug resistance protein 1*-B2 | **GAAGTCGCTTATCATGAGC** CCCTATAGTGAGTCGTATTA |
| *Multidrug resistance protein 1*-B3 | TAATACGACTCACTATAGGG **GAAGTCGCTTATCATGAGC** |
| *Multidrug resistance protein 1*-B4 | **GCTCATGATAAGCGACTTC** CCCTATAGTGAGTCGTATTA |
| *Multidrug resistance protein 1*-C1 | TAATACGACTCACTATAGGG **GAGAGTGAAAAGGTGGTGC** |
| *Multidrug resistance protein 1*-C2 | **GCACCACCTTTTCACTCTC** CCCTATAGTGAGTCGTATTA |
| *Multidrug resistance protein 1*-C3 | TAATACGACTCACTATAGGG **GCACCACCTTTTCACTCTC** |
| *Multidrug resistance protein 1*-C4 | **GAGAGTGAAAAGGTGGTGC** CCCTATAGTGAGTCGTATTA |
| *Probable chitinase 3*-A1 | TAATACGACTCACTATAGGG **GATATGAAGATCCGGACTC** |
| *Probable chitinase 3*-A2 | **GAGTCCGGATCTTCATATC** CCCTATAGTGAGTCGTATTA |
| *Probable chitinase 3*-A3 | TAATACGACTCACTATAGGG **GAGTCCGGATCTTCATATC** |
| *Probable chitinase 3*-A4 | **GATATGAAGATCCGGACTC** CCCTATAGTGAGTCGTATTA |
| *Probable chitinase 3*-B1 | TAATACGACTCACTATAGGG **GCAGCGAAGTCAATCCACC** |
| *Probable chitinase 3*-B2 | **GGTGGATTGACTTCGCTGC** CCCTATAGTGAGTCGTATTA |
| *Probable chitinase 3*-B3 | TAATACGACTCACTATAGGG **GGTGGATTGACTTCGCTGC** |
| *Probable chitinase 3*-B4 | **GCAGCGAAGTCAATCCACC** CCCTATAGTGAGTCGTATTA |
| *Probable chitinase 3*-C1 | TAATACGACTCACTATAGGG **GTTCCCCCACCCCACTGAC** |
| *Probable chitinase 3*-C2 | **GTCAGTGGGGTGGGGGAAC** CCCTATAGTGAGTCGTATTA |
| *Probable chitinase 3*-C3 | TAATACGACTCACTATAGGG **GTCAGTGGGGTGGGGGAAC** |
| *Probable chitinase 3*-C4 | **GTTCCCCCACCCCACTGAC** CCCTATAGTGAGTCGTATTA |
| *Probable chitinase 3*-D1 | TAATACGACTCACTATAGGG **GCGAATCACAAATTCAATC** |
| *Probable chitinase 3*-D2 | **GATTGAATTTGTGATTCGC** CCCTATAGTGAGTCGTATTA |
| *Probable chitinase 3*-D3 | TAATACGACTCACTATAGGG **GATTGAATTTGTGATTCGC** |
| *Probable chitinase 3*-D4 | **GCGAATCACAAATTCAATC** CCCTATAGTGAGTCGTATTA |
| *Probable chitinase 3*-E1 | TAATACGACTCACTATAGGG **GAGAGAATATTTGCCAGCC** |
| *Probable chitinase 3*-E2 | **GGCTGGCAAATATTCTCTC** CCCTATAGTGAGTCGTATTA |
| *Probable chitinase 3*-E3 | TAATACGACTCACTATAGGG **GGCTGGCAAATATTCTCTC** |
| *Probable chitinase 3*-E4 | **GAGAGAATATTTGCCAGCC** CCCTATAGTGAGTCGTATTA |
| *Pupal cuticle protein*-A1 | TAATACGACTCACTATAGGG **GTCTTCTGATGGACACCAC** |
| *Pupal cuticle protein*-A2 | **GTGGTGTCCATCAGAAGAC** CCCTATAGTGAGTCGTATTA |
| *Pupal cuticle protein*-A3 | TAATACGACTCACTATAGGG **GTGGTGTCCATCAGAAGAC** |
| *Pupal cuticle protein*-A4 | **GTCTTCTGATGGACACCAC** CCCTATAGTGAGTCGTATTA |
| *Pupal cuticle protein*-B1 | TAATACGACTCACTATAGGG **GGCTCAAGTTCAGCTGACC** |
| *Pupal cuticle protein*-B2 | **GGTCAGCTGAACTTGAGCC** CCCTATAGTGAGTCGTATTA |
| *Pupal cuticle protein*-B3 | TAATACGACTCACTATAGGG **GGTCAGCTGAACTTGAGCC** |
| *Pupal cuticle protein*-B4 | **GGCTCAAGTTCAGCTGACC** CCCTATAGTGAGTCGTATTA |
| *GFP*-1 | TAATACGACTCACTATAGGG **GAAGCAGCACGACTTCTTC** |
| *GFP* -2 | **GAAGAAGTCGTGCTGCTTC** CCCTATAGTGAGTCGTATTA |
| *GFP* -3 | TAATACGACTCACTATAGGG **GAAGAAGTCGTGCTGCTTC** |
| *GFP* -4 | **GAAGCAGCACGACTTCTTC** CCCTATAGTGAGTCGTATTA |

Note：The letters after each gene indicated different copies of the gene. Number 1 after each gene copy is regarded as top strand for sense, number 2 as bottom strand for sense, number 3 as top strand for antisense, and number 4 as bottom strand for antisense. The sequences in bold are the silencing gene fragment of the interest, and those without bold are the promoters in the synthesis kit.
