# Supplementary material for: Knockdown of Thitarodes host genes influences dimorphic transition of Ophiocordyceps sinensis in the host hemolymph
Source: Front Cell Infect Microbiol. 2024 Sep 27;14:1451628. doi: 10.3389/fcimb.2024.1451628 (PMC11466941; doi:10.3389/fcimb.2024.1451628)
Supplement: Supplementary file 3 [file Table3.doc]

**TABLE S3 |** Primers used for qRT-PCR of the knockdown genes

| Name | Sequence 5’-3’ |
| --- | --- |
| *Cuticle protein 18.6, isoform B* -R | CAATAGTGGTGGTGGGCGG |
| *Cuticle protein 18.6, isoform B*-F | GCGCAAGGTCGACTACTCTG |
| *Ecdysone-induced protein 78C*-F | CGCATCATCCCTATTCCGCT |
| *Ecdysone-induced protein 78C*-R | TGATAATCCTGGGCGTTCGG |
| *Flightin*-F | CGCACATACACCAGCAAGC |
| *Flightin*-R | TGATTTGCTGTGGTAGCGGT |
| *Larval cuticle protein LCP-30*-F | AGCTTCCGGTTATGGTGTCG |
| *Larval cuticle protein LCP-30*-R | ATCCATCGGGAACTTCGTCG |
| *Multidrug resistance protein 1*-F | CCAAAGTTGGCAGGATGTGC |
| *Multidrug resistance protein 1*-R | TGAGTTCTGAGTGCGTTCCC |
| *Probable chitinase 3*-F | ATGTGGGACAGTGCTTCGTT |
| *Probable chitinase 3*-R | ACGCTCTTAGCAGTGGGAAC |
| *Pupal cuticle protein*-F | GTAAGGCCGATTCCGGTCAA |
| *Pupal cuticle protein*-R | GTCCAAGACGTGGTGTCCAT |
| *Translation elongation factor 2*-F | TCGGGAGAACACATCATTGC |
| *Translation elongation factor 2*-R | GGAAGACCGTCGGGCATAG |

Note：F, forward primer; R, reverse primer.
